# Strategy-based evaluation of a formative simulation test assessing professionally relevant competences of undergraduate medical students

**DOI:** 10.3205/zma001745

**Published:** 2025-04-15

**Authors:** Sarah Prediger, Julia Gärtner, Lea Jebram, Sigrid Harendza

**Affiliations:** 1 Universitätsklinikum Hamburg-Eppendorf, III. Medizinische Klinik, Hamburg, Germany

**Keywords:** evaluation, feedback, formative assessment, communication, competences, simulation

## Abstract

**Objectives:**

A strategy-based evaluation of new teaching or examination formats is recommended in order to check their suitability before a possible integration into a medical curriculum. The aim of the project was to evaluate an established formative competence- and simulation-based examination format for medical students in a standardized way.

**Project description:**

In a realistic simulation of a first working day in hospital, medical students at the end of their studies were given the opportunity to test their medical competences and to develop them further on the basis of self-assessment and feedback. In the years 2020 to 2023, a total of 879 students participated. This included 707 students in their final year (PJ) and 172 students before their PJ. The simulation format was evaluated in a standardized manner based on the Stanford criteria for evaluating clinical teaching. The free text comments were analyzed by content using MAXQDA.

**Results:**

The quality of the training, the practical learning content and aspects of the individual training phases are the most important areas of the student evaluation. The learning atmosphere, the leadership and the assessments and feedback received are described positively. There are also clear indications that the simulation format promotes understanding and retention and supports self-directed learning well. Overall, participants gave the teaching format an average school grade of 1.26±.45. Some evaluation results have already been implemented in the adaptation of the simulation.

**Conclusion:**

The integration of the simulation format into medical curricula can be recommended on the basis of this evaluation. Further development of the format is also possible.

## Zusammenfassung

**Zielsetzung:**

Eine strategiebasierte Evaluation von neuen Lehr- oder Prüfungsformaten empfiehlt sich, um deren Eignung vor einer möglichen Integration in ein medizinisches Curriculum zu überprüfen. Das Ziel des Projektes war es, ein etabliertes formatives kompetenz- und simulationsbasiertes Prüfungsformat für Medizinstudierende standardisiert zu evaluieren.

**Projektbeschreibung:**

Medizinstudierenden wurde zum Ende ihres Studiums in einer realitätsnahen Simulation eines ersten Arbeitstags im Krankenhaus die Gelegenheit gegeben, ihre ärztlichen Kompetenzen zu überprüfen und auf der Basis von Selbsteinschätzung und Feedback weiterzuentwickeln. In den Jahren 2020 bis 2023 nahmen insgesamt 879 Studierende teil. Darunter waren 707 Studierende im Praktischen Jahr (PJ) und 172 Studierende vor dem PJ. Das Simulationsformat wurde auf Basis der Stanford-Kriterien zur Evaluation von klinischer Lehre standardisiert ausgewertet. Die Freitextkommentare wurden inhaltsanalytisch mit MAXQDA ausgewertet.

**Ergebnisse:**

Die Qualität des Trainings, die praktischen Lerninhalte und Aspekte der einzelnen Trainingsphasen sind die wichtigsten Themenbereiche der Studierenden-Bewertung. Das Lernklima, die Leitung sowie die erhaltenen Bewertungen und das Feedback werden positiv beschrieben. Es zeigen sich außerdem deutliche Hinweise, dass das Simulationsformat das Verstehen und Behalten fördert und selbstgesteuertes Lernen gut unterstützt. Insgesamt bewerten die Teilnehmenden das Lehrformat im Mittel mit der Schulnote 1,26±,45. Einige Evaluationsergebnisse wurden bereits in der Anpassung der Simulation umgesetzt.

**Schlussfolgerung:**

Die Integration des Simulationsformates in medizinische Curricula kann auf Basis dieser Evaluation empfohlen werden. Eine Weiterentwicklung des Formates ist ebenfalls möglich.

## 1. Introduction

Responsible medical work and action is expected of graduates of human medicine studies upon licensure resp. from the first day of further medical training. This requires the acquisition of competences that are mapped in the National Competence-Based Learning Objectives Catalog for Medicine (NKLM) [https://nklm.de/zend/menu] and should be acquired during the six-year studies. Since competence is the ability to act responsibly and appropriately in a given context by integrating complex knowledge, skills and attitudes [1], [2], simulation formats are suitable for learning and testing competences in order to demonstrate responsible and competent medical action. Before new teaching or examination formats are integrated into a curriculum, it is advisable to first establish them as voluntary offers and evaluate their suitability.

The Federal Ministry of Health’s draft bill, which also provides for “the reorganization of examinations and the introduction of new, modern examination formats” ([3], p.1), is intended, among other things, to better depict communication with patients in history taking and the medical management of patients ([3], p.14). For the final year (PJ), it is also planned to complete a minimum number of structured patient presentations ([3], p.35f.). Furthermore, universities are to be obliged to offer courses that prepare students for the third stage of the medical examination [3].

The overarching aim of the project was, therefore, to evaluate an established format [4] for its suitability of integration into the curriculum, which supports medical students in the acquisition and self-reflection of medical skills based on feedback. To this end, students were given the opportunity to test their medical competences in a realistic simulation of a first working day in hospital [4] and to develop them further on the basis of self-assessment and feedback. The evaluation serves to improve teaching and is, therefore, an inherent part of it [5], whereby teaching quality is a multidimensional construct. In terms of methodology and practice, the evaluation strategy essentially followed the recommendations outlined in the position paper of the GMA committee “teaching evaluation” [6].

## 2. Project description

In the present project, a strategy-based content evaluation based on the Stanford criteria for the evaluation of clinical teaching [7] was carried out for a formative competence-based simulation assessment of a telemedical first working day in hospital [8]. From 2020 to 2023, a total of 879 students took part in the formative simulation assessment. This included 707 students in the PJ and 172 students before the PJ (semesters 6 to 12). In addition, 303 students took part in the simulation format repeatedly. The participants were on average 26.8±3.6 years old and 66.6% were female. Participation was voluntary and places were allocated according to the order of registrations received.

### 2.1. Formative simulation assessment

The formative simulation assessment of a first working day in hospital is based on a validated simulation format [4], which has been carried out as a telemedical format since 2020 [8] and comprises three basic phases:

1. consultation hour,
2. case preparation,
3. case presentation and discussion round with an attending physician.

**Figure 1:**
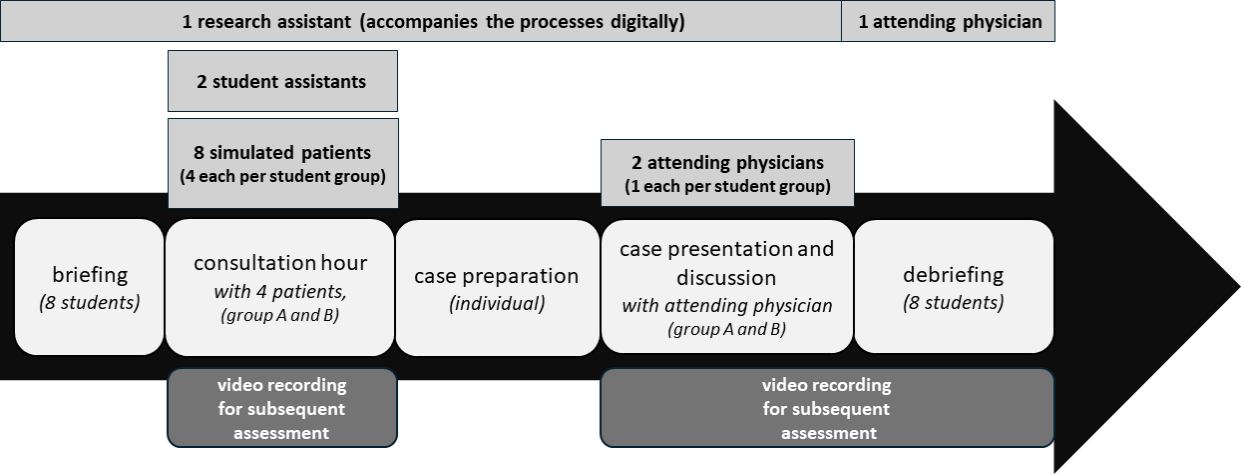
Procedure of the simulation format with human resources

The conversion of the format from face-to-face to digital implementation had no impact on the basic process. The structure of all phases is identical. The patient interviews and group discussions now take place digitally. The only differences are that the students participate from home, i.e. they are physically “alone”, and the aspect of telemedical care for patients has now become part of the simulation (see figure 1 (Fig. 1)).

The simulation was established on the basis of the ten most important, consented competence facets for beginning residents [9]. Some of the competence facets represent general learning objectives across the simulation, while others include specific learning objectives in the three basic phases (see table 1 (Tab. 1)). The other practical learning objectives of the simulation format are practicing focused history taking and case presentation as well as differential diagnostic thinking.

An assessment tool for medical communication and interpersonal skills (ComCare) was validated for the consultation hour [10], which was used by the simulated patients to provide feedback to the students. A digital documentation possibility was created for the management phase. Participants were able to view various patient findings for the handover discussion or case presentation. This was followed by a digital case discussion in groups of four, each with an attending physician, so that – from the students’ perspective – all four clinical pictures of the patients they had seen during the consultation hour were discussed.

The formative simulation assessment offered in the sense of deliberate practice [11] is advertised to the target group of students with the opportunity to test their own competences. Reference is made to the learning gain in terms of the acquisition of “adaptive expertise” [12]. For the participants, the focus is not on the character of an examination, but on the simulation format as training or exercise.

### 2.2. Evaluation approach

An evaluation was carried out at the end of each simulation. The evaluation questionnaire, which was created using LimeSurvey (version 6.5), includes closed questions (1 “strongly disagree” to 5 “strongly agree”) and open-ended free text questions as well as an overall assessment of the simulation format in the form of a school grade.

The free text fields offer the opportunity to provide information on the following aspects:

- aspects that were particularly liked in the training
- reasons for recommending the training to others
- free feedback option for any further thoughts on the training
- reasons for repeated participation (for those who have participated several times)

In this way, it is possible to find out which aspects are particularly important to the participants because they address these themselves and are not only asked closed questions on predefined aspects. The closed questions on aspects of teaching quality complete the evaluation. Together with the open answers, these can be analyzed using the seven Standford criteria for evaluating clinical teaching [7]. Litzelman et al. [13] have formulated the seven Stanford criteria as shown in table 2 (Tab. 2).

The response rate for the evaluation was 98.7%. A total of 603 participants provided information in the free text fields. The total of 918 free text comments were analyzed using MAXQDA Analytics Pro (version 22.6.0). The comments were assigned to the inductively created categories according to their content. These were in turn assigned to the Stanford criteria of good clinical teaching and then further discussed in order to check the learning objectives. The individual comments generally address several statements, so that some of them were assigned to several content categories, which means that the added percentages in table 3 (Tab. 3) add up to more than 100%. The number per category was set in relation to the total number of free text comments in order to get an impression how relevant which aspects were for the participants.

## 3. Results

Table 3 (Tab. 3) shows the free texts according to the inductively generated content categories “Quality of the training”, “practical learning content”, “training phases”, “learning atmosphere”, “acknowledgements”, “learning aspects”, “aspects relating to the patients”, “praise of the organization”, and “receiving feedback”. In general, it can be seen that the quality of the training, the practical learning content and aspects of the individual training phases are the most important topics for students when it comes to evaluation. Aspects of the case discussion with an attending are mentioned most frequently (40.5%) when it comes to describing what was liked best. Practicing and learning clinical reasoning and differential diagnostic, a key learning objective of the simulation, is the most common reason why participants would recommend the simulation format to others (43.7%). Aspects of learning and knowledge acquisition and comparison, which are important aspects of self-directed learning, are the most frequently cited reasons for repeated participation (34.6%).

The participants rated the teaching format overall with an average school grade of 1.26±.45. In the following, the evaluation results are structured according to the seven Stanford criteria listed above and examined in more detail:

### 3.1. Learning atmosphere (criterion 1)

The participants (S1-S23) described a very positive learning atmosphere in the simulation, which apparently led to positive learning outcomes and encouraged repeated participation. A total of 21.1% of the evaluation comments address the positive aspects of the learning atmosphere experienced. The added value lies in this:

*“(…) practice on didactically selected cases without the pressure of the real situation in the hospital.“ (S1)*

Although some of the students report initial fears and concerns about being overwhelmed or similar, in the simulation they experience a *“protected environment”* (S2) in which they can try things out and achieve learning success:

*“It’s like jumping in at the deep end. You're a bit afraid of making a fool of yourself, but nothing can really happen except that you learn a lot.“ (S3)*

Participants also praise the pleasant atmosphere, which allows them to make mistakes:

*“A very good interactive, uncomplicated, practical and exam simulation (...); helpful that it was not overwhelming and that mistakes were not seen as a problem but as an opportunity to learn a systematic approach.“ (S4)*

*“I liked it that the discussion with the attending physicians created an atmosphere in which you were allowed to make mistakes and in which the attending physicians provided additional explanations.“ (S5)*

The fun factor is also highly emphasized. This aspect was explicitly mentioned in 69 comments. The combination of fun during clinical work in the simulation with the perception of a challenging course is also evident:

*“It was a lot of fun to take the patients’ history and to think clinically with the findings sent to me. The case presentation with (...) [the attending physician] was also very interesting! All in all, it was a pretty challenging and good course.“ (S6)*

### 3.2. Leadership (criterion 2)

The participants state that the teachers were able to convey the content of the course well and in a way that was appropriate for the target group (4.70±.51). In the free texts, the good support during the simulation was praised in many cases. Aspects of the evaluation of the didactic concept, which are also frequently mentioned in the comments on what was particularly pleasing, are aspects of general structuring and time structuring, which belong to the Stanford criterion “leadership”. The conceptual planning of the simulation allows it to be effectively managed structurally and the important learning aspects can be perceived in a focused and concentrated manner.

*“Practicing clinical thinking, very good time structuring, working on the case and precise questions from the senior physician. By asking questions and listening to other questions, you are much more actively involved in learning than by just reading.“ (S7)*

By encouraging and challenging, the teaching staff can adapt the simulation to the learning pace of the students. This is made explicit in the final case discussion, in which the attending physicians’ supervision is described very positively:

*“Practice-oriented, good cases, good attending physician who supported you very well in finding the solution yourself.“ (S8)*

The opportunity to work independently is also described very positively:

*“The training was the first opportunity for me to prepare a patient case properly on my own and to initially face the questions and challenges alone, and the learning effect for me was enormous. I also found out where my weaknesses lie and what I need to pay more attention to, all in all really great.“ (S9)*

### 3.3. Communicating goals (criterion 3)

The participants rated the course as didactically well prepared (4.80±.44) and described the achievement of learning objectives that were implied in the various phases of the simulation:

*“In my opinion, the simulation training particularly promotes the taking of medical histories. You have to structure yourself well and not forget anything. (...) The fact that the cases are also followed-up in detail afterwards promotes the learning success.“ (S10)*

*“Knowledge gain, especially with regard to focused history taking and case presentation, practicing free speech in a supervised situation without exam stress.“ (S11)*

*“The cases are didactically great, I am a big fan of the preparation and the passion of the teaching of (...) [the lecturer] and her team and I always take a lot from this training! I'm also currently in the 100-day learning plan, so I'm naturally looking forward to being able to apply my 'knowledge'.“ (S12)*

### 3.4. Promoting understanding resp. comprehension and self-directed learning (criteria 4 and 7)

In addition to achieving the objectives, the participants clearly describe the successful promotion of understanding and retention of the learning content:

*“The simulated patients gave you a realistic feeling and increased your differential diagnostic skills. Looking beyond the typical clinical picture for typical symptoms and considering the entire patient with all laboratory values is something you don’t usually learn at university.“ (S13)*

*“I had memorable and important experiences and gained a lot of motivation and desire for medicine.“ (S14)*

*“Good opportunity to practise history taking, differential diagnostic thinking and patient presentation. Valuable feedback from attending physicians and patients. Gain of information that stays in the mind longer than dull memorization.“ (S15)*

There are also concrete learning experiences that encourage further, self-directed learning:

*“Thank you very much, it was a lot of fun and the learning effect was once again great. It helps enormously to close your own knowledge gaps as well as your own gaps in the medical history taking and to optimize the case presentation to attending physicians!!!” (S16)*

The perceived sense of responsibility (3.99±.91) is also an important learning experience that can prepare for everyday clinical practice in the initially protected space of the simulation. In addition, the simulation also seems to contribute to leaving one's own “comfort zone” (S17) and gaining self-confidence, which is an important aspect of clinical work for taking up responsibility and working in a team:

*“Because you gain self-confidence and realize that you don’t have to know everything. Clinical thinking and consideration are almost more important.“ (S18)*

As described above, the simulation makes it possible to become aware of one’s own knowledge gaps, learning needs and potential for improvement and thus to support sustainable learning and one’s own control of learning:

*“I consider this training to be highly relevant and one of the best ways to check your current level of knowledge under realistic conditions and uncover your own potential for improvement/knowledge gaps.“ (S19)*

Important aspects of self-regulation that arise from self-reflection are revealed and thus activate self-directed learning:

*“The training is particularly good for reflecting on your own deficits. As we never experience exams such as OSCEs with feedback, I (...) take a lot more away from today's training.“ (S20)*

*“The first time I took part, I was noticeably overwhelmed in terms of time management during the history taking. (...) I managed this much better this time. I also feel more confident in the differential diagnostic approach. My future focus will be on a structured case presentation (...). During my clinical clerkships, I only had a few opportunities to do this and I realize that I can benefit enormously from participating.“ (S21)*

### 3.5. Evaluation and feedback (criteria 5 und 6)

Self-directed learning was also stimulated by the assessment and feedback in the simulation. The participants received both quantitative assessments as well as verbal and written feedback during and after the simulation. Both the feedback from the teachers (attending physicians) and the feedback from the simulated patients (4.87±.36 and 4.72±.61, respectively) was perceived as very valuable by the participants. Many also emphasized the importance of feedback in their comments. The professional feedback with “good tips” for clinical thinking is very much appreciated and the tips from the simulated patients are seen as helpful for the further development of communication skills.

*“Because it's great to learn differential diagnostics and the feedback from the attending physician and the patients has a positive influence on my personal development.“ (S22)*

*“Feedback from the patients is also very important to me in order to improve my interviewing skills. I received very helpful feedback last time. I also found practicing case presentations very helpful and especially the tips from (...) [the attending physician] on how to present your case briefly and concisely.“ (S23)*

## 4. Discussion

The results show a very positive acceptance of the simulation format with clear indications for the implementation of good clinical teaching according to the Stanford criteria and the fulfillment of the learning objectives. The positive perception of the opportunity to work independently is an important learning experience for the participants, especially in view of the fact that this is rarely experienced in this form during their studies and also in the PJ. This also demonstrates the successful integration of the general learning objective of taking responsibility. The space for independence mentioned by the participants, which is perceived as “free” but still functions in the sense of “guided discovery” [14] in the simulation, shows the aspect of successful leadership (criterion 2) and is perceived positively by the participants. In this process of guided discovery, the participants deal with problems (history taking and case preparation) and receive advice (examination findings, support during the case discussion) and feedback (during the case discussion and after the simulation) from the teachers. This approach thus enables students to reflect on their own work process under the guidance of the teacher [15]. This allows the participants to engage in a so-called productive effort, i.e. a challenge in which mistakes can also be made and for which the strongest association with learning success could be shown [16]. A learning atmosphere that is experienced as positive (criterion 1) is an essential component of successful learning [17]. The fun experienced in this not only ensures that the learning environment is perceived positively, but can also contribute to better learning [18], [19]. The learning atmosphere, which is experienced as pleasant and allows mistakes to be made, shows the potential to develop the competence 'dealing with mistakes' in the simulation. The combination of fun, learning challenge and positive learning atmosphere, together with the feedback, create a good balance in the overall learning environment of the simulation, leading to a positive learning experience and the achievement of learning goals in terms of acquiring “adaptive expertise” [12]. Newly acquired motivation and the reports of repeatedly participating persons show that the learning content is sustainably internalized and thus has a consistency that shapes learning, understanding and retention (criterion 4) and that students make sustainable use of the opportunity for self-directed learning (criterion 7).

As participation was voluntary, it cannot be ruled out that particularly motivated students with a positive attitude towards the simulation format took part, which could have had an influence on the evaluation results. In addition, the evaluation was focused on the Stanford criteria, which could have led to other equally important aspects not being sufficiently highlighted. On the other hand, the evaluation data of 868 participants could be analyzed in a standardized way with the help of these Stanford criteria. As required for evaluations [6], various consequences at the structural and procedural level of the simulation format can be derived from the evaluation results. Adjustments to the format were already made during the project as a result of the evaluation findings. For example, an additional training phase has been carried out since 2022, in which students discuss the cases in small groups in advance before case presentation. This corresponds to everyday working life and leads to better achievement of the learning objectives. Further options for adapting the simulation are continuously reviewed on the basis of the evaluation.

## 5. Conclusion

Based on the standardized evaluation carried out, the integration of the simulation format described here into the curriculum can be recommended. Further development of the format is also possible on this basis. In addition, the simulation format can be used longitudinally and evaluated from this perspective over time.

## Notes

### Ethics

This project was carried out in accordance with the Declaration of Helsinki. The Ethics Committee of the Hamburg Medical Association approved this project (PV3649). Participation in the formative simulation assessment was voluntary. Written informed consent was obtained.

### Funding

This work was supported by the Joachim Herz Stiftung and the Medical Faculty of Hamburg University.

### Authors’ ORCIDs

- Sarah Prediger: [0000-0001-5483-1983]
- Sigrid Harendza: [0000-0002-7920-8431]

## Acknowledgements

We would like to thank all participants for taking part in the evaluation.

## Competing interests

The authors declare that they have no competing interests. 

## Figures and Tables

**Table 1:**
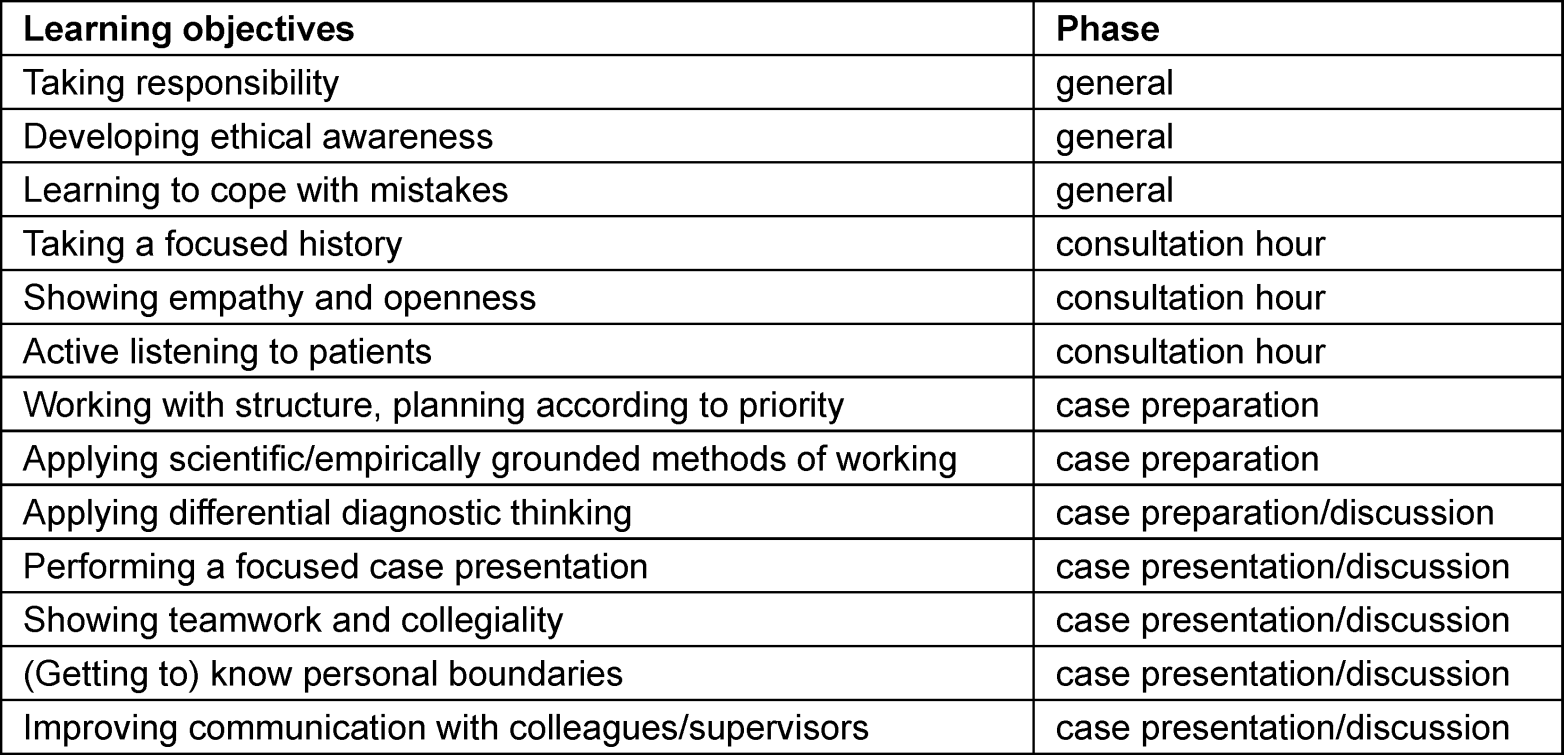
Learning objectives in different competence areas according to simulation phases

**Table 2:**
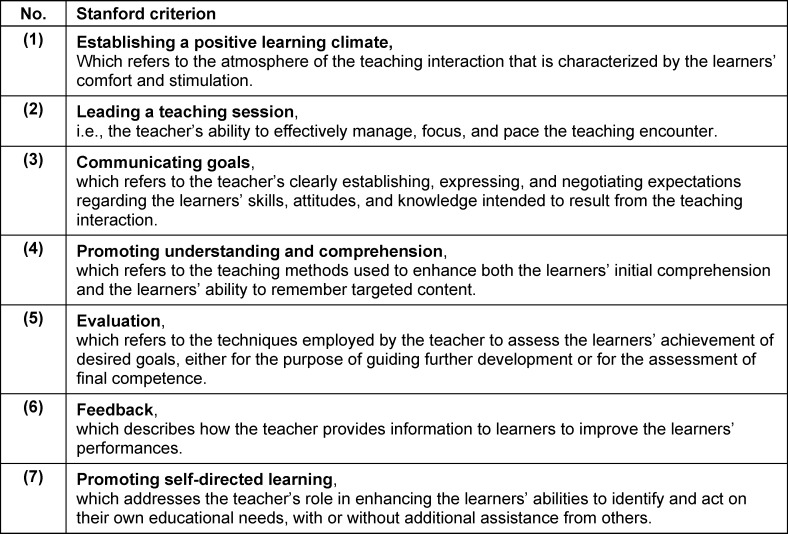
Formulation of the Stanford criteria according to Litzelman et al. [13]

**Table 3:**
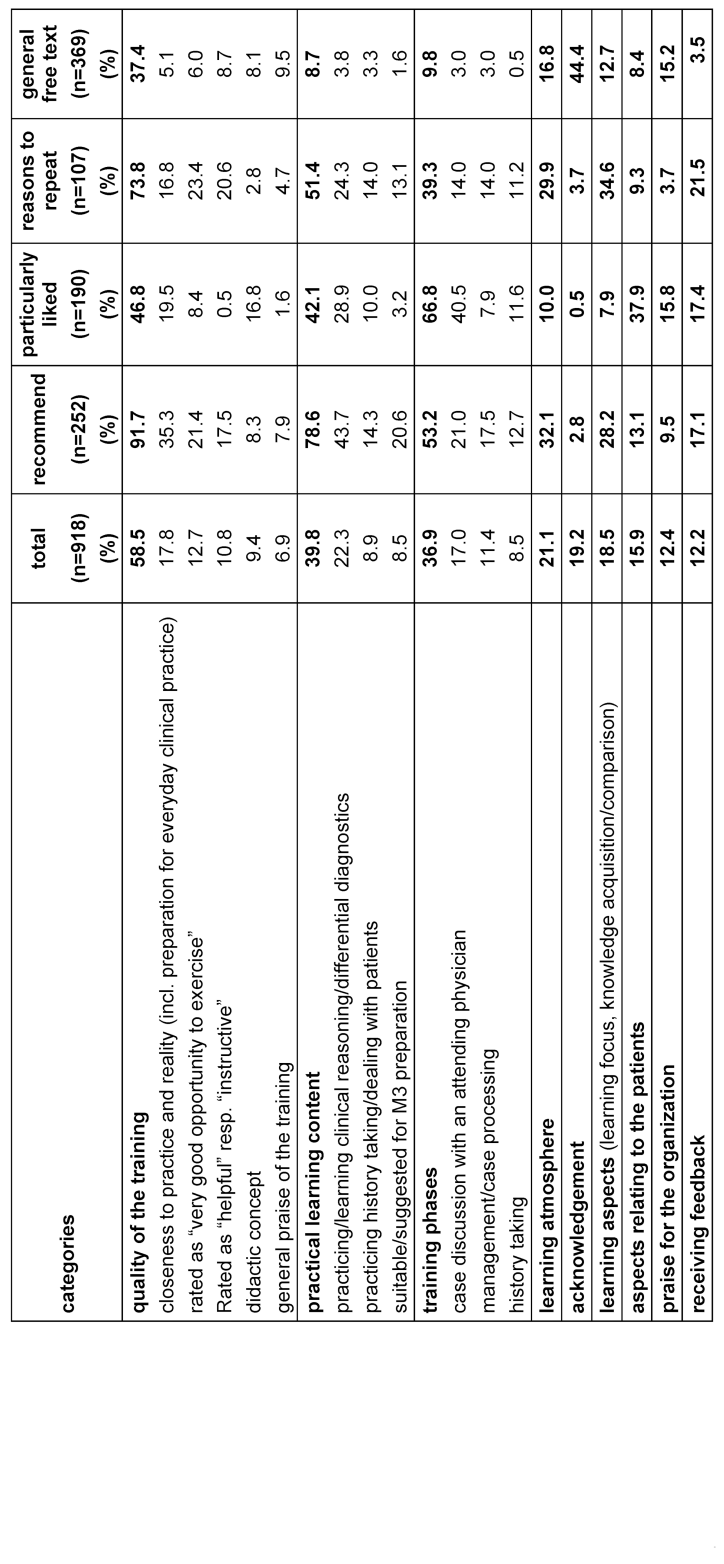
Evaluation results by category and frequency
